# Life stress and Bipolar Disorder: regarding a clinical case

**DOI:** 10.1192/j.eurpsy.2023.1490

**Published:** 2023-07-19

**Authors:** T. Coelho Rocha, J. F. Cunha, S. Torres, J. Alves Leal, J. Carvalho Moura, D. Seabra, I. Monteiro Lopes, A. Lopes, G. Lima

**Affiliations:** Psychiatry and Mental Health Department, Centro Hospitalar Barreiro Montijo, Barreiro, Portugal

## Abstract

**Introduction:**

Research on life stress in bipolar disorder largely fails to account for the possibility of a dynamic relationship between psychosocial stress and episode initiation. The kindling hypothesis states that over the course of recurrent affective disorders, there is a weakening temporal relationship between major life stress and episode initiation that could reflect either a progressive sensitization or progressive autonomy to life stress.

**Objectives:**

To explore the concept of the Kindling model applied to bipolar disorder and to present a clinical case of a bipolar patient whose latter mood episodes were caused by adverse life events.

**Methods:**

We performed a non-systematic literature review using the most relevant papers found on the database PubMed with the keywords “kindling effect”, “allostatic load”, “bipolar disorder” and “prevention”. Description of the clinical case report.

**Results:**

The phenomenon of kindling was first discovered by Goddard in 1967 who described it in epilepsy. Later, Post applied it to the bipolar disorder, arguing that the initial episodes of both unipolar and bipolar affective disorders are often precipitated by psychosocial stressors, but after multiple recurrences, not only do precipitated episodes continue to occur, but so do spontaneous ones as well. We present the case report of a 62 years old woman, divorced, diagnosed with type 1 bipolar disorder since she was 20 years old. She always have had poor adherence to her medication and follow-up with Psychiatry consultation, with a non-containing sociofamily environment that does not promote clinical stability. Over the time, her admissions on the Psychiatry ward were more frequent and precipitated by adverse life events, mainly caused by the deteriorated relationship with her children.

**Conclusions:**

The kindling model clarifies aspects of the longitudinal course of episode development, recurrence, and progression to spontaneity, as well as further conceptual and theoretical rationales for intervention in order to prevent illness progression.

**Disclosure of Interest:**

None Declared

